# Anterior Hox Genes and the Process of Cephalization

**DOI:** 10.3389/fcell.2021.718175

**Published:** 2021-08-05

**Authors:** James C.-G. Hombría, Mar García-Ferrés, Carlos Sánchez-Higueras

**Affiliations:** Centro Andaluz de Biología del Desarrollo (Consejo Superior de Investigaciones Científicas/Junta de Andalucía/Universidad Pablo de Olavide), Seville, Spain

**Keywords:** Hox genes, cephalogenesis, evolution, arthropods, vertebrates

## Abstract

During evolution, bilateral animals have experienced a progressive process of cephalization with the anterior concentration of nervous tissue, sensory organs and the appearance of dedicated feeding structures surrounding the mouth. Cephalization has been achieved by the specialization of the unsegmented anterior end of the body (the acron) and the sequential recruitment to the head of adjacent anterior segments. Here we review the key developmental contribution of Hox1–5 genes to the formation of cephalic structures in vertebrates and arthropods and discuss how this evolved. The appearance of Hox cephalic genes preceded the evolution of a highly specialized head in both groups, indicating that Hox gene involvement in the control of cephalic structures was acquired independently during the evolution of vertebrates and invertebrates to regulate the genes required for head innovation.

## Evolutionary Relationships of Cephalic Hox Genes

Hox genes are found in almost all animals, generally organized in large clusters of up to 15 genes. Their amino acid sequence has been used to classify Hox proteins in distinct homology groups and to infer how the cluster evolved (Zhang and Nei, 1996). Comparative analyses indicate that well developed clusters comprising at least seven Hox genes were already present more than 550 million years ago, suggesting their evolution was concurrent with the diversification of the main animal body plans that appeared during the Cambrian explosion (de Rosa et al., 1999).

**FIGURE 1:**
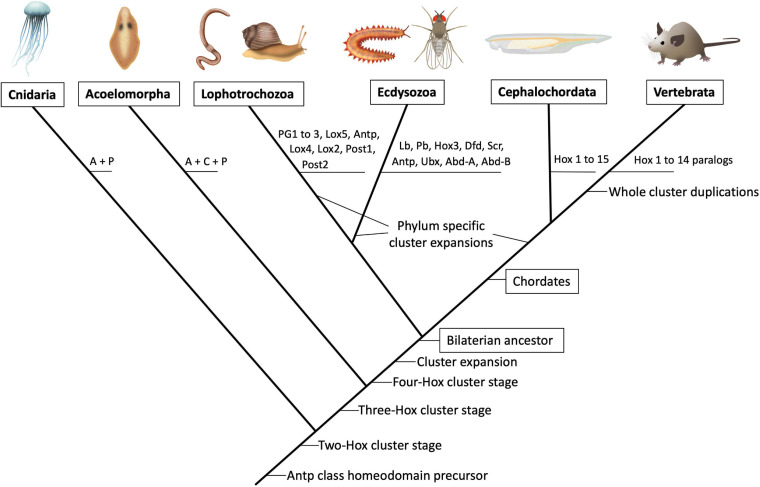
Schematic phylogenetic tree showing the predicted evolution of the Hox paralogous groups. Illustrations of major extant animal groups are presented on top joined by their currently accepted branching points (not to temporal scale). The predicted appearance of particular Hox paralog gene precursors is depicted on the branching points. In the vertebrate lineage the whole cluster duplicated several times (not represented) giving rise to four paralogous Hox clusters named HoxA to HoxD in birds and mammals. In teleost fish a further duplication gave rise to eight Hox clusters. For a comprehensive analysis of Hox cluster evolution (see Figure 2A). A, anterior; C, central; P, posterior.

The comparison of Hox complexes among living animal groups allows inferring the cluster’s temporal evolution. No Hox genes have been found in simple animal forms like Sponges, Ctenophores, and Placozoa (Biscotti et al., 2014) but are present in Cnidarians (jellyfish and sea anemones), where they also control axial development (He et al., 2018). Cnidarian Hox genes are only related to the Anterior and the Posterior Hox groups, suggesting the Cnidarians diverged from other animals at an early stage in the complex’s expansion (Figure 1). Slightly more diverse Hox complexes are present in the Acoels (extremely simple wormlike creatures), which probably constitute a sister group to all other bilaterians (Achatz et al., 2013). Acoels possess Hox proteins related to the Anterior, Central and Posterior paralogy groups. In contrast, all other animals studied to date have expanded clusters containing between 8 and 15 Hox genes that belong to seven defined groups (Biscotti et al., 2014). These include complex animals like the Chordates (which include the vertebrates), the Lophotrochozoa (which include the annelid worms and the molluscs); and the Ecdysozoa (which include the arthropods and the Onychophora).

**FIGURE 2:**
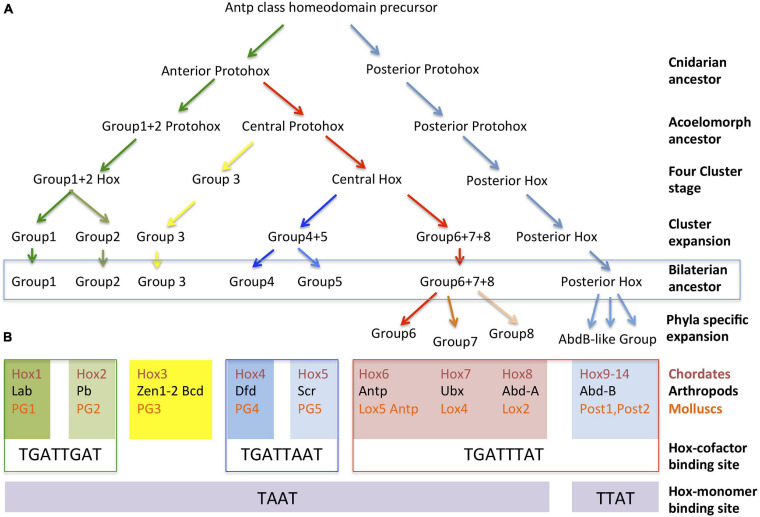
Evolution of Hox protein clusters and correlation with their DNA binding preferences. **(A)** Inferred cluster evolution from sequential tandem duplications of an ancient Antp class homeobox gene. Three tandem duplications gave rise to a four-gene ProtoHox cluster. Duplication of the full ProtoHox cluster gave rise to the ParaHox cluster (not shown) and the Hox cluster proper. The duplication of specific genes in the four-gene Hox cluster resulted in its expansion in the common ancestor that gave rise to all bilateral animals. Phyla specific duplications resulted in the final expansion that resulted in the 10–15 Hox clusters found in modern species. **(B)** Classification of the Chordate, Arthropod, and Mollusc Hox proteins into paralogy groups according to their evolutionary relationships as inferred from their sequence similarities. Each of the seven groups is aligned over their preferred DNA binding sites either when in complex with TALE cofactors or as monomers. For simplicity, the Hox6-Hox8 genes have been organized in columns aligned with particular arthropod and mollusc equivalents, even though direct homology for genes in the column has not been demonstrated, see text.

Several hypotheses based on sequence similarities have been proposed to explain how the Hox cluster expanded from a single original Antennapeadia class homeobox protein. This precursor protein duplicated to form an initial ProtoHox cluster composed of an Anterior and a Posterior gene (Figure 2A). After two consecutive duplications, or unequal crossovers, the Anterior Hox gave rise to new genes (Gehring et al., 2009). This resulted in a three gene-cluster composed of an Anterior, a Central and a Posterior Hox gene and then to a four gene-cluster encoding an Anterior, Group 3, Central and Posterior Hox. Sequence similarities with other non-Hox Antennapedia-class homeodomain proteins indicate that the ProtoHox gene cluster duplicated at some point during this initial expansion giving rise to the ParaHox cluster and the Hox cluster proper (Garcia-Fernandez, 2005a,b).

All known Hox genes can be related to one of these early four groups, suggesting that multiple tandem duplications expanded this primitive four Hox cluster prior to the divergence of the bilaterian animals. The duplication of the Anterior gene created the ancestors of Hox1/Labial/PG1 and the Hox2/Proboscipaedia/PG2 homologs in chordates, arthropods and molluscs, respectively (Figure 2B). Group three Hox proteins are represented today by a single Hox3/Hox3/PG3 homolog, although in insects this gene lost its typical Hox expression and got involved in extra-embryonic membrane specification and the establishment of the maternal antero-posterior axis (Zen, Zen2, and Bicoid) (Falciani et al., 1996; Hughes et al., 2004). A stepwise duplication of the Central gene gave rise to Hox4–8/Dfd, Scr, Antp, Ubx, abd-A/Antp, Lox5,4,2 (de Rosa et al., 1999; Li et al., 2020). Orthologs across species are difficult to assign for some of the Central group Hox genes. Central group sequence comparisons only cluster reliably Hox4 with Dfd and Hox5 with Scr, leaving uncertainty about when the Hox6–8/Antp, Ubx, abdA/Lox5, Antp, Lox4, Lox2 precursors formed. Thus, these must have arisen by independent duplications after the Chordate, Lophotrocozoa, and Ecdysozoa ancestors diverged or, if present before that time, their sequence has diverged so much that now it is impossible to confidently assign them to specific groups (Zhang and Nei, 1996; Hueber et al., 2010). Finally, duplication of the posterior gene created the ancestors of Hox9–14/Abd-B/Post 1–2.

In summary, the available data indicate that the Hox1–5 paralogy groups, that are expressed in cephalic regions, were already present in the bilaterian common ancestor of Chordates, Lophotrocozoa and Ecdysozoa, which may have resulted in these proteins sharing functional characteristics among these highly diverse phyla.

## DNA Binding Preferences in Cephalic Hox Proteins

Hox proteins can bind DNA as monomers or in complex with cofactor proteins. Two cofactor proteins are conserved and have been studied in vertebrates (Pbx and Meis) and in *Drosophila* (Exd and Hth). These cofactors contain an atypical DNA-binding homeodomain with a three aminio acid loop extension (TALE) that gives name to their class. Recent analyses have revealed that Hox proteins in complex with their cofactors show distinguishing conserved DNA binding specific preferences.

When binding to DNA as monomers, several studies have shown that Hox proteins recognize the same core sequence, TAAT, except the posterior AbdB-like Hox proteins which favor TTAT (Ekker et al., 1994; Noyes et al., 2008). Finding such a small DNA binding site and the fact that most Hox proteins bound the same sequence was at odds with their known *in vivo* regulatory specificity. Further analyses found that Hox proteins increase their target specificity using two alternative strategies: the use of clustered monomeric Hox sites, and the binding to DNA in complex with TALE cofactors. Hox-cofactor DNA binding results in the enlargement of the DNA recognition site and the increase in DNA binding affinity (van Dijk and Murre, 1994; Ryoo et al., 1999).

A thorough SELEX-seq analysis in *Drosophila melanogaster* of all possible Hox paralog complexes with Extradenticle (Exd) and Homothorax (Hth) has revealed that cofactor interaction uncovers a latent specificity present in the Hox protein that modulates its DNA binding preferences. Hox-cofactor DNA sequence preferences can be used to classify Hox proteins into three classes. Class 1 includes the Labial and Proboscipaedia proteins that present a higher affinity for TGATTGAT; Class 2 includes Dfd and Scr with preferential affinity for TGATTAAT; and Class 3 includes Antp, Ubx, Abd-A, and Abd-B whose preferred binding site is TGATTTAT (Slattery et al., 2011; Figure 2B). Human HoxA1, HoxA5, and HoxA9 when in complex with the homologous vertebrate TALE cofactors have the same *in vitro* binding preferences as the *Drosophila* paralogs (Kribelbauer et al., 2017). In *Drosophila*, these *in vitro* differential binding preferences translate into a differential spatial specific downstream target activation, which can be also replicated by the *Amphioxus* Hox proteins (Sanchez-Higueras et al., 2019). Besides high affinity sites, cephalic Hox proteins may also activate their targets through low affinity binding sites and in some cases even share identical targets or functions with other Hox proteins (Hirth et al., 2001; Kribelbauer et al., 2019; Sanchez-Higueras et al., 2019). Despite this, not only the Hox protein sequence, but also the conservation of high affinity DNA binding preferences across distant species (Figure 2B) sets apart cephalic Hox proteins from other Hox proteins, underlying their ancient evolutionary relationship.

## Hox Protein Characteristics Influencing Paralog DNA Binding Specificity

Hox proteins present four regions with significant amino acid conservation: the hexapeptide, the linker region, the homeodomain and the C-terminal sequence adjacent to the homeodomain.

The *homeodomain* is the most conserved region. Although other transcription factors also possess homeodomains, four specific amino acids are common to all Hox proteins and probably confer the specific binding properties that distinguish this protein class from other homeodomain containing proteins. Besides the general Hox amino acids, a few amino acids are found to be present in specific paralogous groups providing them with specific characteristics (reviewed in Merabet et al., 2009). The so-called *hexapeptide* is a four to six amino acid sequence N-terminal to the homeodomain and separated from it by the linker region. The hexapeptide (HX) domain mediates the protein interaction with the TALE cofactors (Chang et al., 1995), although in some Hox proteins additional domains have also been found to establish physical Hox-Pbx interactions (Dard et al., 2018; Saurin et al., 2018). The *linker region* separates the HX from the homeodomain. It has a variable length that in many species has been shown to correlate with the paralogous group (In der Rieden et al., 2004; Merabet et al., 2009). The *C-terminal region* confers specific characteristics to paralogy group 1 Hox genes and can exert important regulatory functions (see below).

Although very few paralog specific characteristics have been studied in detail, some have been uncovered at the molecular level for the cephalic Hox1 and Hox5 proteins. The Labial/Hox1 paralog presents two particularities, the first one is that the hexapeptide, besides interacting with the Pbx/Exd co-factors, as in other Hox proteins, also has an inhibitory effect on the homeodomain preventing Lab/Hox1 binding to DNA. Inhibition is released when the Exd cofactor binds to the HX allowing the homeodomain to bind DNA (Chan et al., 1996). Whether Labial may still operate fully independent of Exd *in vivo* is still unclear. A second particularity has been found by cross-species functional analysis of Labial and its mouse orthologs HoxA1 and HoxB1. These studies uncovered how a six amino acid motif called CTM (C-terminal motif), located C-terminal to the homeodomain, modulates the Hox1-Pbx physical-interaction mediated by the HX. Although sequence conservation between HoxA1 and Lab is only about 30%, expression of the vertebrate protein can fully rescue fly *labial* mutant defects, indicating an ancestral function conserved by both paralogs. The conserved CTM motif present in both Labial and HoxA1 is required to retain an optimal physical interaction with Exd/Pbx1 as well as to perform the ancestral *in vivo* target regulation (Singh et al., 2020). In contrast, the HoxB1 paralog that has a divergent CTM sequence cannot rescue all *labial* mutant phenotypes in the fly. The divergent CTM motif reduces HoxB1′s interaction with Pbx1, preventing the formation of a ternary Hox-Pbx-Meis complex, which results in a different repertoire of genomic targets *in vivo.* This has been proposed to represent a case of Hox paralog neo-functionalization in brain and head tissues during development (Singh et al., 2020). *In vitro* SELEX-seq analysis of human HoxA1/Pbx shows similar DNA binding site preferences as its *Drosophila* counterpart (Kribelbauer et al., 2017). Additional structural and biochemical studies will help to decipher whether the CTM’s modulatory effect on HoxB1-Pbx interactions either creates a new range of binding preferences, or forces Labial to operate preferentially as a monomer in a context-dependent manner.

Another case of paralog specificity has been described for the Scr/Hox5 paralog group, where it has been found that the interaction of Scr with their main DNA binding cofactor (Exd/Pbx) is not only necessary to recognize the DNA sequence, but also the specific DNA shape of its target DNA backbone, which has a strong impact on both DNA binding strength and specificity. A combination of structural studies, biochemistry and *in vivo* assays in *D. melanogaster* embryos showed that the specific binding of Scr-Exd heterodimers to a target site (AGATTAATCG) in the *fkh250* enhancer relies on the optimal interaction with the specific minor groove conformation. The width of the minor groove is determined by the DNA sequence, that in this case creates a narrowing of the groove in which two key Scr paralog-conserved basic amino acids (Arg3 and Arg5) from the homeodomain’s N-terminal arm motif “RQR” and a Histydine from the adjacent linker region (His12) specifically interact (Joshi et al., 2007). These Hox-DNA interactions at the binding site’s minor groove do not involve hydrogen bonds, unlike the direct homeodomain’s third helix recognition of the bases in the major groove, and cannot take place without the Exd-YPWM interaction (Joshi et al., 2007; Slattery et al., 2011). Thus, it has been proposed that the binding preference of Dfd/Scr-Exd complexes for Class II core sites (TGATTAAT) exhibited in SELEX-seq experiments is strongly influenced by the conserved “RQR” motif and linker region (Joshi et al., 2007; Slattery et al., 2011). Moreover, high-throughput analyses including SELEX-seq as well as *in vivo* experiments in embryos showed that mutations of those Scr amino acids selecting DNA shape, bias the Scr-Exd binding preferences toward different core motifs including Class I (TGATTGAT) and Class III (TGATTTAT) (Abe et al., 2015). Interestingly, it was also shown that Scr-Exd DNA binding specificity can be transferred to an Antp-Exd complex by mutating the residues from the N-terminal arm and linker to those involved in minor groove width recognition in Scr (Abe et al., 2015). Therefore, Hox-Exd complexes can discriminate differences in DNA minor groove shape through a reduced number of key side chain amino acids to establish a different set of functional binding specificities *in vivo*.

## Function and Expression of Vertebrate Cephalic Hox Proteins

The functional analysis of vertebrate Hox genes is complicated by the existence of several paralogous Hox clusters due to successive duplications of the ancient Hox chordate cluster (Holland et al., 1994). In birds and mammals there are four Hox paralogous clusters named HoxA to HoxD containing paralogous genes that, in many cases, have redundant functions. Despite this, mutational analyses have demonstrated that vertebrate Hox proteins also control the morphological differentiation of repeated metameric structures along the antero-posterior axis. As in insects, spatial Hox expression in partially non-overlapping regions along the anterior-posterior body axis is key to confer each metamere with segment specific structures.

Experimental studies in mice and chick embryos, have shown Hox genes are required for the correct antero-posterior body axis segmental specification in the neural tissue, the branchial arch derivatives and the axial skeleton (Mallo et al., 2010; Philippidou and Dasen, 2013; Parker and Krumlauf, 2020). The requirement of Hox function is especially clear during embryonic development when studying the formation of the hindbrain rhombomeres, the neural crest cells and the somites, three structures with a transient segmental organization during early development.

### Cephalic Hox Genes and Hindbrain Development

**FIGURE 3:**
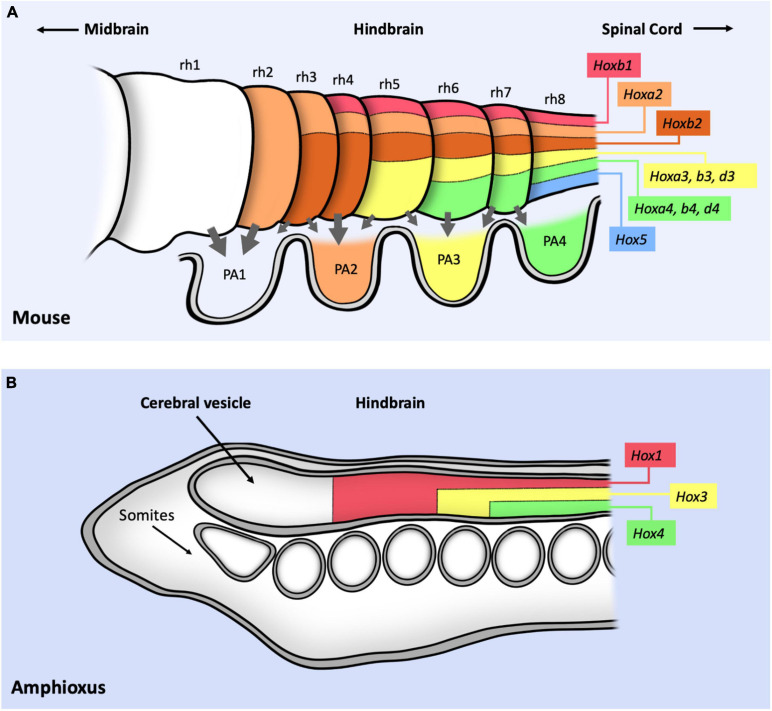
Schematic representation of Hox expression in the cephalic mouse and Amphioxus central nervous systems. **(A)** Summary of Hox1–5 expression in the hindbrain, labeling the position of the eight different rhombomeres (rh1–8). Gray arrows represent the migratory movement of the cranial neural crest cells toward the pharyngeal arches (PA1–4). PA are colored following the pharyngeal Hox code: no Hox proteins are expressed in PA1; HoxA2 and B2 proteins are expressed from PA2 to PA4; HoxA3, B3 and D3 from PA3 to PA4; HoxA4, B4, D4 and Hox5 in PA4. **(B)** Expression of three Hox genes in the anterior part of the *Amphioxus* CNS. Note that the conserved relative position of Hox gene expression with respect to the mouse CNS suggest a positional correlation of both nervous systems despite the lack of rhombomeres or neural crest cells in *Amphioxus*. In both panels, information only concerns antero-posterior expression and not dorso-ventral expression. Figure based on data from references (Holland et al., 2008; Philippidou and Dasen, 2013; Parker et al., 2018).

The hindbrain is the more posterior region of the vertebrate brain, giving rise to the pons, the medulla and the cerebellum. During early development the hindbrain becomes subdivided into eight segments known as rhombomeres (rh) that constitute lineage-restricted groups of cells that do not intermix (Figure 3A). Major nerves arise from different rhombomeres. Cephalic Hox genes are required for both hindbrain segmentation and for the specification of the motor nerves originating from the rhombomeres. In mice, abnormal rhombomeric segmentation is observed in mutations for Hox1 and Hox2 paralogy groups. Although single mutation of *Hoxa1* or *Hoxa2* genes already affect rhombomere segmentation, these defects increase in double mutants. Compound *Hoxa1/Hoxb1* mutants lack both rh4 and rh5 (Gavalas et al., 1998; Studer et al., 1998; Rossel and Capecchi, 1999). Compound *Hoxa2/Hoxb2* mutants lack boundaries between rh1 and rh4 (Davenne et al., 1999), and *Hoxa1/Hoxa2* double mutants completely lack rhombomere boundaries (Barrow et al., 2000).

The formation of specific hindbrain motoneurons also depends on cephalic Hox gene function. In mutants for *Hoxa2* the motoneurons of the trigeminal nerve, which forms in rh2–3, are disorganized and misrouted (Gavalas et al., 1997) while ectopic expression of *Hoxa2* in rh1 generates a trigeminal-like nerve (Jungbluth et al., 1999). In *Hoxb1* mutants, facial nerve motoneurons arising from rh4 acquire the characteristics of the trigeminal nerve leading to the loss of the facial nerve (Goddard et al., 1996; Studer et al., 1996; Gavalas et al., 2003). Moreover, ectopic expression of *Hoxb1* in rh1 can generate facial-like motoneurons, while its expression in rh2 transforms the trigeminal neurons into facial neurons (Bell et al., 1999). The abducens nerve originating in rh5 is absent in *Hoxa3/Hoxb3* double mutants and ectopic *Hoxa3* can induce its formation (Gaufo et al., 2003; Guidato et al., 2003). Although these results show the importance of Hox genes in the formation of particular nerve types, the cross-regulatory interactions among Hox gene expression and the requirement of more than one paralogous Hox group for nerve specification complicates the analysis.

### Cephalic Hox Genes and Neural Crest Derivatives Development

The neural crest cells originate during development in different antero-posterior positions of the dorsal neural tube including the diencephalon, the mesencephalon, the rhombencephalon (the hindbrain) and the spinal cord. These cells lose their epithelial character and become migratory, giving rise to a variety of structures including cartilage, bones, pigment cells, peripheral neurons or glia depending on the segment where they are formed. The cranial neural crest cells (cNCC) originate from the anterior neural tube (Figure 3A, gray arrows), specified by Otx2 and the Hox1–4 genes (Minoux and Rijli, 2010). cNCC originating from the hindbrain migrate in separate streams to colonize the pharyngeal arches (PA). These cells participate in the formation of the ventral cranial bones and the nerve ganglia, and influence the migratory routes of the motoneurons growing from the rhombomeres. Cranial neural crest cells can give rise to cartilage while trunk neural crest cells do not. The cNCC originating from rh1 do not express any Hox gene while those from more posterior rhombomeres express Hox1–5 paralogs. *Hoxb1* (and *Hoxb2*) is expressed in rh4 and in the second pharyngeal arch (PA2). *Hoxa2* is active in rh3 and rh5 and in the neural crest cells colonizing PA2-A4. *Hoxa3* is expressed in rh5-rh6 (and *Hoxb3* is expressed in rh6-rh8) colonizing PA3 and PA4. *Hoxd4* is expressed in PA4 (Minoux and Rijli, 2010) (Figure 3A). *Hox5* is expressed in rh8 (Philippidou and Dasen, 2013) and at PA4 (Holland and Hogan, 1988; Kam and Lui, 2015). Mutation of the Hox1 paralog group results in the absence of all rh4 derived neural crest cells (Gavalas et al., 2001; McNulty et al., 2005). Mutation of the Hox2 paralogous genes result in the transformation of the PA2 derivatives into structures normally formed by PA1 in a typical homeotic transformation (Gendron-Maguire et al., 1993; Rijli et al., 1993; Hunter and Prince, 2002; Santagati et al., 2005). Ectopic Hox2 paralog gene expression in PA1 derivatives cause their transformation into structures normally formed by PA2 (Grammatopoulos et al., 2000; Pasqualetti et al., 2000; Hunter and Prince, 2002; Kitazawa et al., 2015). Inactivation of Hox3 paralogs cause malformations of the PA3 and PA4 skeletal derivatives although these are not homeotic transformations (Condie and Capecchi, 1994; Manley and Capecchi, 1997). Interestingly, deletion of the HoxA cluster in cNCCs causes a partial homeotic transformation of PA3 and PA4 derivatives into PA1-like structures, indicating a requirement for both *Hoxa2* and *Hoxa3* (Minoux et al., 2009). Despite advances on our knowledge on Hox expression and function on the neural crest cells it is still unclear how the Hox genes integrate with the neural crest gene network (Parker and Krumlauf, 2020).

### Cephalic Hox Genes and Axial Skeletal Development

Although not specifically cephalic structures, the cephalic Hox genes contribute to the specification of the cervical vertebrae during the development of the most anterior axial skeleton. The somites are transient embryonic structures produced by the segmental organization of the presomitic mesoderm that appear at both sides of the nerve cord prefiguring the axial skeleton. Besides the axial skeleton, somites give rise to several structures including dermis, tendons and muscles. The vertebrae originate from the ventral part of the somite, which experiences an Epithelial to Mesenchymal Transition (EMT) giving rise to the sclerotome.

*Hoxa3/Hoxd3* are required for the development of the most anterior vertebrae: the atlas and the axis. Some Hox3 paralog mutants present homeotic transformations as well as defects that could be due to lack of vertebrae primordia cell proliferation. This defect is stronger in double *Hoxa3/Hoxd3* mutations, which present a complete deletion of the atlas (Condie and Capecchi, 1994). Loss of Hox4 paralogs cause transformations of cervical vertebrae (C) toward their anterior counterparts, the atlas and the axis. In triple *Hoxa4/b4/d4* mutants, morphological characteristics of the atlas appear in C2–C5 vertebrae as well as some defects in C6 and C7 which resemble Hox5 mutant phenotypes (Horan et al., 1995).

Hox5 affects both the development of posterior cervical vertebrae and of anterior thoracic vertebrae and ribs (McIntyre et al., 2007). Hox5 mutants show transformations of the vertebrae toward the C2 (the axis). In this respect, Hox5 presents similar phenotypes to Hox6 mutants although Hox6 affect only from C7 to posterior segments. Ectopic Hox6 induces ectopic ribs in the cervical and lumbar regions.

## Function and Expression of Arthropod Cephalic Hox Proteins

**FIGURE 4:**
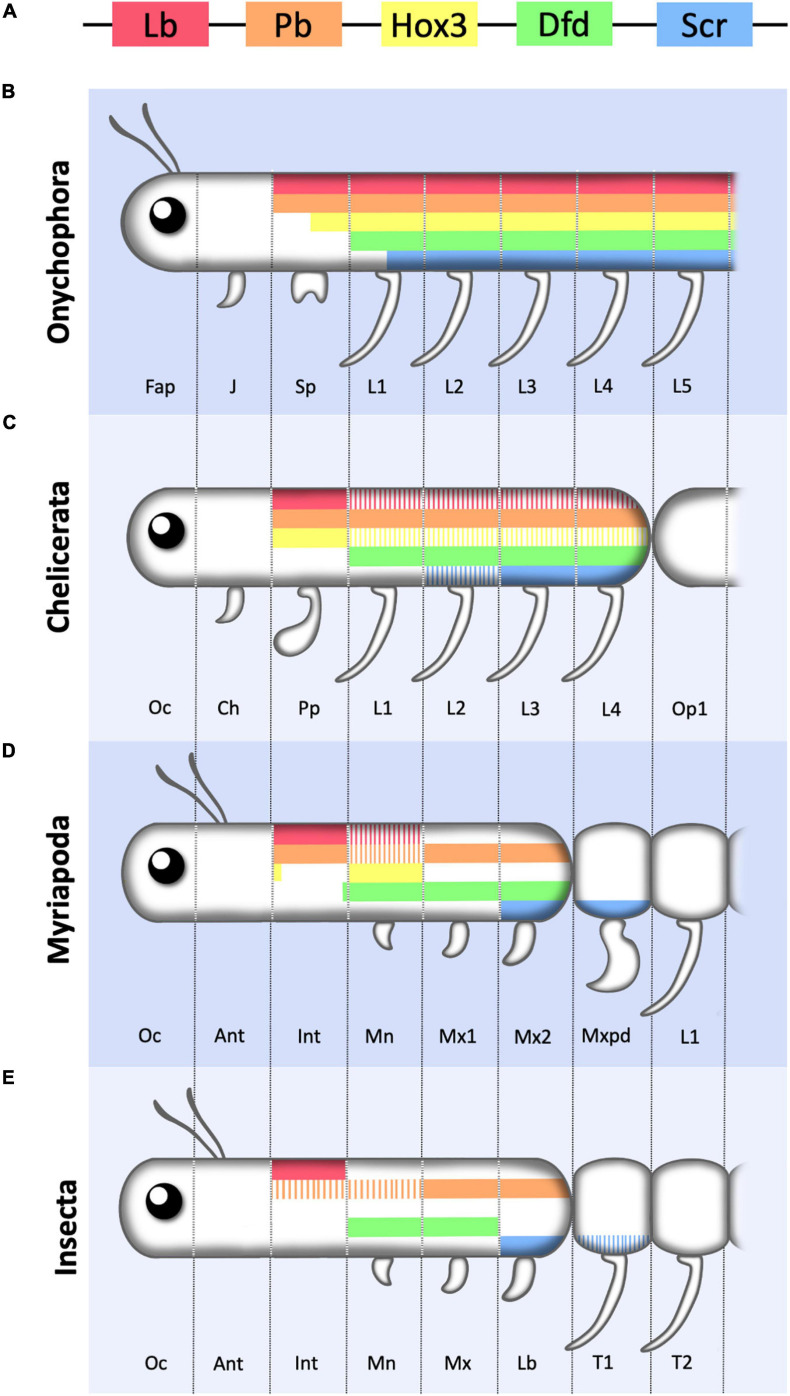
Schematic representation of Hox1–5 expression in the anterior region of the Onychophora and various arthropods. **(A)** Color key of the five Hox genes considered in this review represented following the relative positions they occupy in the cluster. **(B)** Onychophora showing the three head segments (Fap, frontal appendage; J, jaw; Sp, slime papilla) and the first five leg (L) bearing segments. **(C)** Chelicerate showing the first three cephalic segments (Oc, ocular; Ch, chelicerae; Pp, pedipalp) and the four legged prosomal segments. **(D)** Myriapod showing the six cephalic segments (Oc, ocular; Ant, antennal; Int, intercalary; Mn, Mandibula; Mx1, first maxilla; Mx2, second maxilla) followed by the trunk leg (L) bearing segments of which the most anterior one forms a maxilliped (Mxpd). **(E)** Insect showing the six cephalic segments (Oc, ocular; Ant, antennal; Int, intercalary; Mn, Mandibula; Mx, maxilla; Lb, Labium) followed by two of the three thoracic leg (L) bearing segments. Hox3 has not been represented as in most insects it has lost its Hox function. Note that the most anterior cephalic segments lack Hox expression, and that anterior Hox genes required for the formation of cephalic structures in insects may be expressed in leg bearing segments in other phyla, indicating that there is no strict correlation between cephalization vs. trunk development and Hox1–5 expression. For simplicity Crustaceans are not included in the figure and coloring represents antero-posterior Hox expression only. Solid bars represent main or stronger expression compared to striped bars. Figure based on data from references (Hughes and Kaufman, 2002b; Janssen et al., 2014).

Four Hox genes are expressed in the cephalic segments of *Drosophila*: *lab*, *pb*, *Dfd*, and *Scr* which are homologous to Hox1, Hox2, Hox4, and Hox5. The Hox3 gene homolog has lost its homeotic function in insects and acquired new functions in the specification of the extra-embryonic membranes and in anterior maternal specification. However, in other arthropods Hox3 expression fits with a homeotic activity (Figure 4).

The head of *Drosophila*, as that of other insects, is composed by a non-segmented region or acron followed by six segments, three pre-oral (labral, antennal, and intercalary) and three post-oral (mandibula, maxilla, and labium) (Juergens and Hartenstein, 1993). The acron, the labrum and antennal segments do not express any Hox gene although their development requires other homeobox containing genes like *otd*, *ems*, and *btd*, homologs of which are also used in vertebrates for the development of the most anterior head structures, suggesting a deep conservation of the anterior head’s organization (Hirth et al., 2003).

*labial* (*lab*) is the Hox gene expressed most anteriorly, becoming activated in the intercalary segment, also known as tritocerebral segment (Hughes and Kaufman, 2002b). Embryonic *lab* loss of function in *Drosophila* results in larval phenotypes such as head involution defects and the absence of the H piece, but no homeotic transformations. Adult hypomorphs or mitotic clones show various head defects. In the anterior region of the head, there is a deletion of the vibrissae and the maxillary palps, and in the posterior region of the head, a transformation toward thoracic-like bristles and the appearance of thoracic spiracles, suggesting a transformation toward the mesothoracic segment (Diederich et al., 1989; Merrill et al., 1989). In pedipalp bearing arthropods, like spiders, *lab* disruption induces appendage loss from the tritocerebral segment (Pechmann et al., 2015). Several studies have implicated *lab* as an essential neuronal regulator. The tritocerebral neuromere, which corresponds to the most posterior part of the arthropod brain, is severely affected in *lab* mutants, showing loss of neuronal markers and axonal patterning defects (Hirth et al., 1998). Interestingly, this can be rescued by ectopic supply of any other Hox gene, except *Abd-B*, indicating that *cis*-regulatory elements confer the specificity of the interaction, rather than the Hox protein (Hirth et al., 2001). The role of *lab* in larval neuronal control is less explored nonetheless. Kuert et al. (2012) proposed that during the transition to the third larval instar (L3), *lab* induces apoptosis on two specific neuroblast lineages. By blocking apoptosis, they were able to rescue these two neuroblasts in L3, which are Lab positive.

*proboscipedia* (*pb*) embryonic expression in arthropods is highly variable, spanning from the pedipalp segment to the fourth leg segment in chelicerates to just half of the second antennal segment in crustaceans (Hughes and Kaufman, 2002b). Instead, among insects Pb expression seems to be conserved (Denell et al., 1996; Rogers and Kaufman, 1997). In *Drosophila* embryos, Pb expression in the gnathal segments is dependent on *Deformed* (*Dfd*) and *Sex combs reduced* (*Scr*) (Rusch and Kaufman, 2000), but *pb* null mutants show no apparent functional role during embryogenesis (Pultz et al., 1988). In contrast, adult *pb* null mutants show transformed labial palps into legs (Kaufman, 1978). Conversely, ectopic Pb expression in the leg primordia transforms legs into maxillary or labial palps (Aplin and Kaufman, 1997). In *Drosophila*, it has been proposed that Pb is a competence factor allowing Scr to switch from a T1 function into a proboscis function (Percival-Smith et al., 2013).

*Deformed* (*Dfd*) is expressed in the mandibular and maxillary segments of all arthropods (Hughes and Kaufman, 2002b). As shown by Lohmann et al. (2002), Dfd shapes the mandibular and maxillary boundary by controlling directly the proapoptotic gene *reape*r. In *Dfd* mutants, the border between the two gnathal segments is lost, and this can be rescued by restoring Reaper expression. In *Dfd* mutants, the mouth hooks and the sensory cirri do not develop and the maxillary sensory organ is disorganized. However, some of these defects could be caused indirectly by the defective head involution movements caused by these mutations. Other mouth parts are abnormal in *Dfd* mutants with a possible duplication of the cephalopharingeal plates (Regulski et al., 1987). The different developmental outcomes of Dfd activity in the mandibular and the maxillary segments have been attributed to the modulation of Dfd function in the mandible exerted by the Cap-n-collar (Cnc) protein. Isoform C of the Cnc basic leucine zipper protein in the mandible modulates the transcriptional regulation exerted by Dfd in that segment. In *cnc* null alleles mouth hooks and cirri, which are typical maxillary structures appear in the mandible with the disappearance of certain mandibular structures (McGinnis et al., 1998; Veraksa et al., 2000).

*Dfd* is also involved in neuronal specification, in a similar way to *lab*. Disruption of either one of them induces defects in axonal patterning, indicating that both of them play a role in the establishment of regional neuromere characteristics (Hirth et al., 1998). A specific pathway has been described, where *Dfd* controls autonomously the specification of maxillary neuroblasts by induction of the cell adhesion protein Amalgam. This pathway is redundant with a non-autonomous one controlled by *lab* and *Antp* (Becker et al., 2016).

Cephalic neuronal and endocrine specification can be translated into the control of specific behaviors, like feeding or molting. Friedrich et al. (2016) have shown that Dfd is expressed in the subesophageal ganglion, which innervates the muscles that control food intake. They observed that *Dfd* is required for the specification and maintenance of the feeding unit by regulating the synaptic stability protein Ankyrin2-XL.

*Dfd* is able to control endocrine primordia fate, by promoting the specification of the *Corpora Allata* (*CA*) in the maxilla (Sanchez-Higueras et al., 2014). The *CA* synthesizes juvenile hormone, which promotes the maintenance of the larval stage after molting (Hartenstein, 2006). Interestingly, in the larval termite soldier, *Dfd* seems to be able to respond to juvenile hormone levels to control mandible elongation by activating the *dachshund* transcription factor (Sugime et al., 2019). This indicates a reversed control mechanism where *Dfd* becomes downstream of the mechanisms it activated during embryogenesis.

*Sex combs reduced* (*Scr*) is expressed both in the last cephalic and in the first thoracic segments, a characteristic also observed for Hox5 in vertebrates. Embryonic Scr expression usually occupies the labial segment (the last cephalic segment) and the first thoracic segment (Hughes and Kaufman, 2002b). Similar to *Dfd*, *Scr* is implicated in endocrine organ formation during embryogenesis through the specification of the prothoracic gland (PG) primordia (Sanchez-Higueras et al., 2014). The PG synthesizes Ecdysone, which promotes the transition between larval stages or induces metamorphosis depending on the presence or absence of Juvenile Hormone (reviewed in Hartenstein, 2006). In *Drosophila*, Dfd and Scr are expressed transiently in the endocrine primordia where, together with STAT, mediate expression of the *snail* transcription factor to induce an EMT of these cells necessary for *CA* and PG development (Sanchez-Higueras et al., 2014). In the hemipteran insect bug *Oncopeltus fasciatus*, RNAi against *Scr* disrupts prothoracic gland fate, indicating that this gene network is conserved across insects (Hanna and Popadic, 2020). A recent study points out that Scr is activated in *Bombyx mori* larval prothoracic glands, where it negatively regulates the levels of Ecdysone to control the number of molts (Daimon et al., 2021). This suggests that the same circuit used to specify glands during embryogenesis is recruited at later stages to modulate endocrine gland function.

Besides the PG, the specification of the salivary glands in the labium also requires transient expression of *Scr*. Scr target genes in the salivary glands include the *fork head*, *sage* and *CrebA* transcription factors (Panzer et al., 1992; Andrew et al., 1994; Abrams and Andrew, 2005; Abrams et al., 2006). A similar control mechanism was proposed to mediate silk gland specification in *Bombyx mori* (Kokubo et al., 1997).

As mentioned before, Scr expression pattern is not restricted to the cephalic tagma, as it is also activated on the first thoracic (T1) segment. RNAi against *Scr* in *Oncopeltus fasciatus* is able to induce a small ectopic wing in T1, which is transcriptionally different from T2 wings. This data indicate that *Scr* could have an ancient function in the repression of wings (Medved et al., 2015).

## Head Evolution and Hox Genes

As described above, Hox genes play an important role in the formation of the posterior cephalic segments in both vertebrates and arthropods. However, comparative analysis of cephalization among species that diverged early in evolution from vertebrates and arthropods, indicates that their ancestors already had a cluster containing the Hox1–5 genes before the formation of a complex head (Holland et al., 2008; Eriksson et al., 2010; Janssen et al., 2014). Due to the convergent recruitment of Hox proteins into cephalization after the split of both lineages, it is unlikely that they regulate similar downstream targets or gene networks in vertebrates and invertebrates. The fact that vertebrate Hox proteins can rescue the phenotypes caused by mutations in the homologous *Drosophila* Hox genes may be due to their capability of occupying similar binding sites present in the target genes of the other species, rather than the target genes themselves being the same.

*Amphioxus*, a chordate that separated from the lineage that gave rise to vertebrates about 500 million years ago is believed to have a similar body plan as their common ancestor (Garcia-Fernandez et al., 2009). *Amphioxus* has a simple nervous system composed of a cerebral vesicle followed by a nerve cord without any rhombomeric subdivisions (Figure 3B). Gene expression analysis shows that *Amphioxus* Hox genes are expressed in the anterior-posterior axis in a very similar relative position to that of their mouse homologs (Figure 3A). This spatial expression conservation allows comparing both CNSs despite their different morphologies, leading to the suggestion that the *Amphioxus* frontal cerebral vesicle corresponds to the vertebrate forebrain and that the hindbrain equivalent of *Amphioxus* has no rhombomeric subdivisions although it has a similar antero-posterior expression of Hox1–4 genes (Holland et al., 2008). Thus, the Hox1–4 spatial gene expression was set out in the primitive chordate nervous system before the evolution of rhombomeres or neural crest cells, implying that these *Hox* genes were recruited later during vertebrate evolution to regulate novel functions that increased cephalic complexity.

A very similar conclusion is reached when studying Hox expression and cephalization in evolutionary distant groups related to the arthropods. Onychophorans, the sister group of the Arthropods, are animals with a relatively simple head despite possessing a full set of cephalic Hox1–5 genes (Janssen et al., 2014; Mayer et al., 2015). The Onychophoran head consists of only three segments, contrasting with the six head segments present in insects, myriapods and crustaceans (Eriksson et al., 2010; Figure 4). In the Onychophorans, each cephalic segment has a modified limb structure which, from anterior to posterior give rise to an antenna, a jaw and a slime papilla. Posterior to the head, a trunk is formed by repeated metameres with a pair of legs of similar shape in each segment. The most anteriorly expressed Hox genes are *pb* and *lab* which are expressed from the slime papilla segment to the most posterior trunk segment; *Hox3* is expressed from the last few cells of the papilla segment posteriorly; and *Dfd* and *Scr* are expressed from the first leg segment backward (Janssen et al., 2014). All other Hox genes are expressed in progressively more posterior trunk segments. Therefore, compared to insects, in Onychophorans only Lab and Pb are expressed in segments with a distinct cephalic character, while all other Hox genes are expressed in segments with a similar trunk external shape. This suggests that *Hox* gene expression in the anterior segments of the animal, especially *Dfd* and *Scr*, predates their involvement in cephalogenesis and, thus, they must have been recruited later in evolution to contribute to the formation of arthropod specific head structures. This prediction fits with the morphological diversity observed between spiders and other arthropods where *Hox* genes like *Dfd* and *Scr*, that contribute to head morphology in centipedes, crustaceans and insects, are expressed in leg bearing segments in the spiders (Damen et al., 1998; Hughes and Kaufman, 2002a; Schwager et al., 2007; Khadjeh et al., 2012).

An interesting observation has been made in centipedes, which lack a differentiated abdomen and have a large trunk made of externally similar segments. The head in these animals is composed of six segments (ocular, antennal, intercalary, mandibular, maxillary I and maxillary II) with the two most anterior ones lacking Hox expression. *labial* and *Proboscipedia* are expressed in the intercalary segment, *Hox3* is expressed mostly in the mandibular segment, *Dfd* is expressed from the mandible to the maxillary II and *Scr* in Maxillary II and the first trunk segment (Hughes and Kaufman, 2002a). Interestingly, the leg appendages in this first trunk segment have diverged in shape from those present in the rest of the trunk, having converted into poison containing fangs. This segment, called the maxilliped, due to its intermediate head and trunk morphology, expresses *Scr* and *Antp*. Although no functional data are available, this pattern of expression suggests that both genes control its novel morphology and thus, in centipedes, a novel cephalic segment may be in the process of being recruited to the head and with it *Antp*, a Hox gene that usually has trunk functions.

In summary, current studies suggest that an extended *Hox* gene cluster had already evolved in a primitive bilateral ancestor and that these genes were probably expressed differentially along the antero-posterior axis in a rather undifferentiated trunk. As the cephalic region evolved, it became more complex by sequentially adding adjacent trunk segments to the primitive head instead of duplicating existing cephalic segments. As a result, the *Hox* genes expressed in the recruited segments were adopted as the key transcriptional regulators modulating the expression of target genes that gave rise to the phylum specific cephalic structures. In both arthropods and vertebrates, anterior Hox genes were used to specify cephalic structures just because they were expressed differentially in the recruited segments adjacent to the primitive head. The fact that anterior Hox proteins had different DNA binding site preferences to those of posterior ones, probably facilitated the differential modulation of target genes in the head versus those activated in the trunk by more posteriorly expressed Hox proteins. Convergent cephalization occurring at different speeds in arthropods and vertebrates may have resulted in different numbers of anterior Hox genes been recruited to cephalic structures. Genes like Hox5 have a very marginal function in the mammalian hindbrain specification, while its Scr homolog is well established as a cephalic gene in insects, even though it is still involved in trunk development. This suggests that any other Hox gene could have been recruited to perform cephalic functions, a process that might still be occurring as is suggested by the recent evolution of a maxilliped segment with intermediate head-trunk morphology in the centipedes. Here an extra seventh cephalic segment could be evolving by recruiting Antp to the head, whilst in most animals paralog group 6 proteins are exclusively involved in trunk development.

## Author Contributions

JH researched, wrote, and organized the manuscript. MG-F and CS-H researched and wrote the manuscript. All authors contributed to the article and approved the submitted version.

## Conflict of Interest

The authors declare that the research was conducted in the absence of any commercial or financial relationships that could be construed as a potential conflict of interest.

## Publisher’s Note

All claims expressed in this article are solely those of the authors and do not necessarily represent those of their affiliated organizations, or those of the publisher, the editors and the reviewers. Any product that may be evaluated in this article, or claim that may be made by its manufacturer, is not guaranteed or endorsed by the publisher.
